# Determining the thermal characteristics of breast cancer based on high-resolution infrared imaging, 3D breast scans, and magnetic resonance imaging

**DOI:** 10.1038/s41598-020-66926-6

**Published:** 2020-06-22

**Authors:** Adolfo Lozano, Jody C. Hayes, Lindsay M. Compton, Jamasp Azarnoosh, Fatemeh Hassanipour

**Affiliations:** 1 Department of Mechanical Engineering, The University of Texas at Dallas, 800 W Campbell Rd ECW-31, Richardson, TX 75080 USA; 2 Raytheon Space and Airborne Systems, 13510 N Central Expy MS 212, Dallas, TX 75243 USA; 3 Department of Radiology, The University of Texas Southwestern Medical Center, 5323 Harry Hines Blvd MC 8896, Dallas, TX 75390 USA

**Keywords:** Cancer imaging, Breast cancer, Mechanical engineering

## Abstract

For over the three decades, various researchers have aimed to construct a thermal (or bioheat) model of breast cancer, but these models have mostly lacked clinical data. The present study developed a computational thermal model of breast cancer based on high-resolution infrared (IR) images, real three-dimensional (3D) breast surface geometries, and internal tumor definition of a female subject histologically diagnosed with breast cancer. A state-of-the-art IR camera recorded IR images of the subject’s breasts, a 3D scanner recorded surface geometries, and standard diagnostic imaging procedures provided tumor sizes and spatial locations within the breast. The study estimated the thermal characteristics of the subject’s triple negative breast cancer by calibrating the model to the subject’s clinical data. Constrained by empirical blood perfusion rates, metabolic heat generation rates reached as high as 2.0E04 W/m^3^ for normal breast tissue and ranged between 1.0E05–1.2E06 W/m^3^ for cancerous breast tissue. Results were specific to the subject’s unique breast cancer molecular subtype, stage, and lesion size and may be applicable to similar aggressive cases. Prior modeling efforts are briefly surveyed, clinical data collected are presented, and finally thermal modeling results are presented and discussed.

## Introduction

In the context of breast cancer screening and diagnosis, infrared (IR) imaging, also referred to as breast thermography or digital infrared thermal imaging (DITI), is an imaging technique where IR images are taken of a patient’s breasts, referred to as “thermograms.” When used adjunctively in a screening or diagnostic environment, IR images are assessed for thermal abnormalities potentially indicating breast cancer. The method is based on the thermographic detection of cancer at the skin resulting from pathophysiologic changes within the breast caused by cancer; these changes include the metabolic and vascular changes associated with cancer development^1^. Lozano and Hassanipour provided a detailed and objective survey of prior clinical studies, both favorable and unfavorable, regarding IR breast thermography^2^. Owing to mixed clinical findings over the past six decades, the United States Food and Drug Administration (FDA) approved IR thermography in 1982 only as an adjunct to other screening modalities (e.g., mammography and ultrasound)^3^. Today, the FDA warns that IR thermography is not a substitute for mammography and should not be used as an isolated diagnostic procedure^4,5^. The primary motivation for using IR thermography has been the fact that IR imaging does not expose the patient to any amount of ionizing radiation, contrary to mammography.

The present study addresses a two-fold, coupled problem: The lack of knowledge of the thermal characteristics of breast cancer and the lack of real clinical data use in the computational thermal modeling of breast cancer, each of which are discussed in detail. First, data regarding the thermal characteristics of cancer, like the metabolic heat generation rate (a critical thermal modeling parameter), are scant and limited in the literature. In fact, the metabolic heat generation rate of living tissue (normal or cancerous) has been the most elusive term to quantify in any bioheat formulation^6^. In 1980, Gautherie provided the most extensive (and only) data available to date regarding the metabolic heat generation rate of cancerous breast tissue^7^. Gautherie reported an empirical relationship between the metabolic heat generation rate of a breast carcinoma and its volume-doubling time. Gautherie observed a hyperbolic relationship between these two parameters based on data from 84 breast cancer patients with cancerous growth sizes ranging between 0.9–3.8 cm and doubling times of 49–676 days. Gautherie offered an approximate range for the metabolic heat generation rate of breast cancer: 4,800–65,000 W/m^3^. No other data have been reported in the literature for this parameter. Absent of any other data, prior breast cancer modeling efforts have historically relied on Gautherie’s relation when modeling the breast with cancer.

Second, with regard to the computational thermal modeling of breast cancer, approximately 30 efforts have attempted to construct a numerical thermal model of the human female breast with cancer. Some researchers have focused on the “forward problem,” i.e., solving the governing equation to determine the surface temperature distribution of the breast with an internal tumor of various sizes and locations. These models have mostly been three-dimensional^8–25^, while others have been two-dimensional, which limited their applicability^26–29^. Other researchers have constructed thermal models for the purpose of solving the “inverse problem,” i.e., determining tumor parameters (e.g., size, location) and tissue thermophysical properties based on surface temperature solutions. These efforts implemented inverse algorithms and artificial neural networks to estimate the tumor location and depth based on the surface temperature distribution from the numerical model. These models have been three-dimensional^30–39^, while others have been two-dimensional^40–44^.

However, of these approximately 30 efforts, only a few prior models have been constructed using experimental data (here, clinical IR images). Ng and Sudharsan matched their steady-state numerical model with real IR images from 3 female subjects; a mannequin of brassiere size 34 C was used for the breast geometry, and the tumor size and location was approximated^10–13,45^. Gonzalez used COMSOL to build a hemispherical breast model and matched surface temperatures for 20 breast cancer patients by varying the tumor’s metabolic heat generation in the model^20^. Bezerra *et al*. used Gambit/Fluent to match surface temperatures with IR images for at least one patient^18,19,33,38^. Most recently, Recinella *et al*. used IR images of subjects in the prone position to match surface temperatures derived from Gonzalez-Hernandez *et al*.’s model^25,46^.

While these efforts in the past have aimed to develop a thermal model of the breast with cancer, the majority have been academic exercises in simulation. These modeling efforts have collectively had the following limitations:

1. The lack of clinical data with which to calibrate the model (here, the thermography of the breast with cancer).
2. The lack of real breast shapes (i.e., surface geometries), which are unique and vary per individual.
3. The lack of real tumor definition (i.e., size and (x, y, z) spatial location within the breast).
4. The use of Pennes’ bioheat equation as the governing equation, which inherently has limitations and simplifications.
5. The lack of real internal vasculature and blood flow field (i.e., hemodynamic) information in the breast.

Therefore, the goal of the present study was to determine the thermal characteristics of breast cancer by constructing a computational thermal (or bioheat) model calibrated to real clinical data. Specifically, the study aimed to quantify the thermal characteristics—i.e., blood perfusion rate, ${\dot{\omega }}_{b}$, and metabolic heat generation rate, ${\dot{Q}}_{m}^{{\rm{{\prime} }}{\rm{{\prime} }}{\rm{{\prime} }}}$—of normal and cancerous breast tissues that best matched surface temperatures observed on IR images. Clinical data consisted of high-resolution IR images, three-dimensional (3D) breast surface geometries, and internal tumor definition from a female subject histologically diagnosed with breast cancer. A state-of-the-art IR camera recorded IR images, a 3D scanner recorded breast surface geometries, and magnetic resonance (MR) imaging data provided tumor definition such as size and (x,y,z) spatial location. 3D scans and tumor definition served as geometric inputs to the model, whereas IR images were used to calibrate the model. This study is distinguished from prior thermal modeling efforts by its use of clinical data to construct and calibrate the model. The present study aimed to address the first three limitations noted above.

## Results

**Figure 1:**
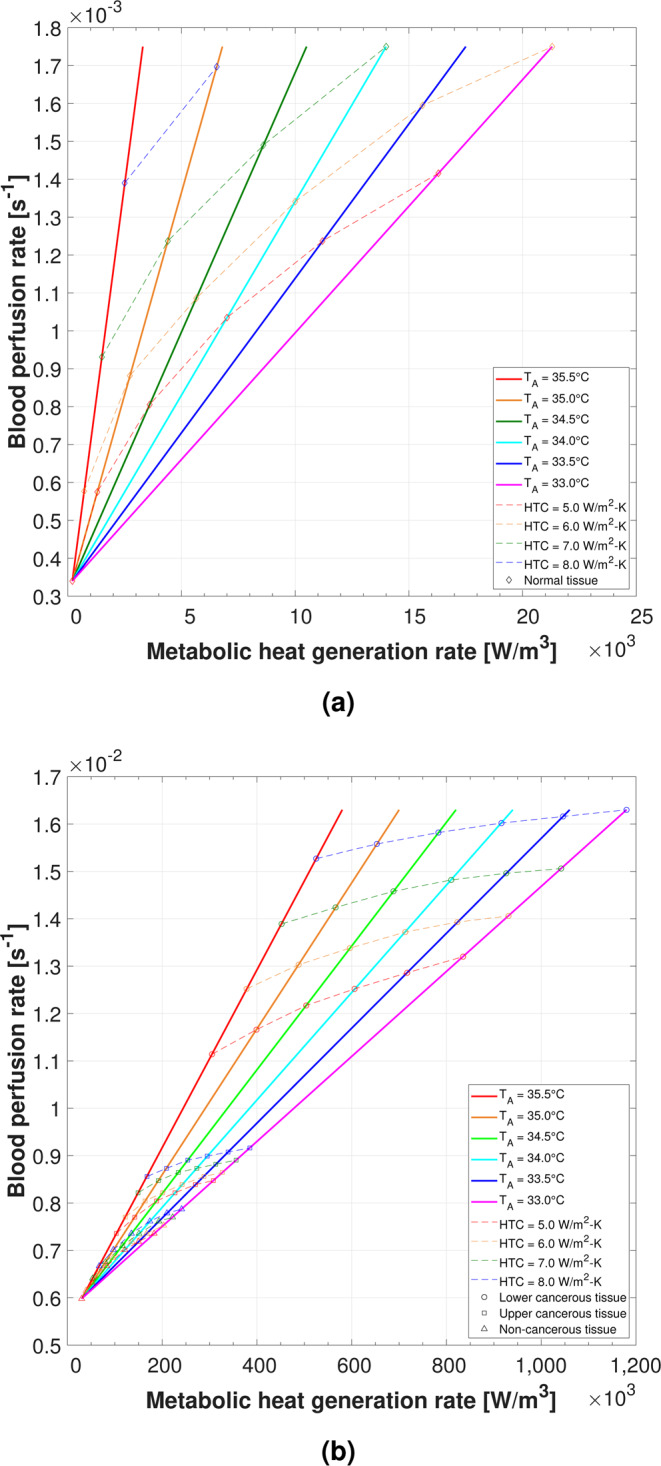
All (${\dot{Q}}_{m}^{{\rm{{\prime} }}{\rm{{\prime} }}{\rm{{\prime} }}},{\dot{\omega }}_{b}$) solutions found in the right normal breast model (**a**) and left malignant breast model (**b**) for varying HTC and ${T}_{A}$. The higher ranges of ${\dot{Q}}_{m}^{{\rm{{\prime} }}{\rm{{\prime} }}{\rm{{\prime} }}}$ for cancerous tissue in each ${T}_{A}$ series were required to reach the nipple hotspot surface temperatures.

**Figure 2:**
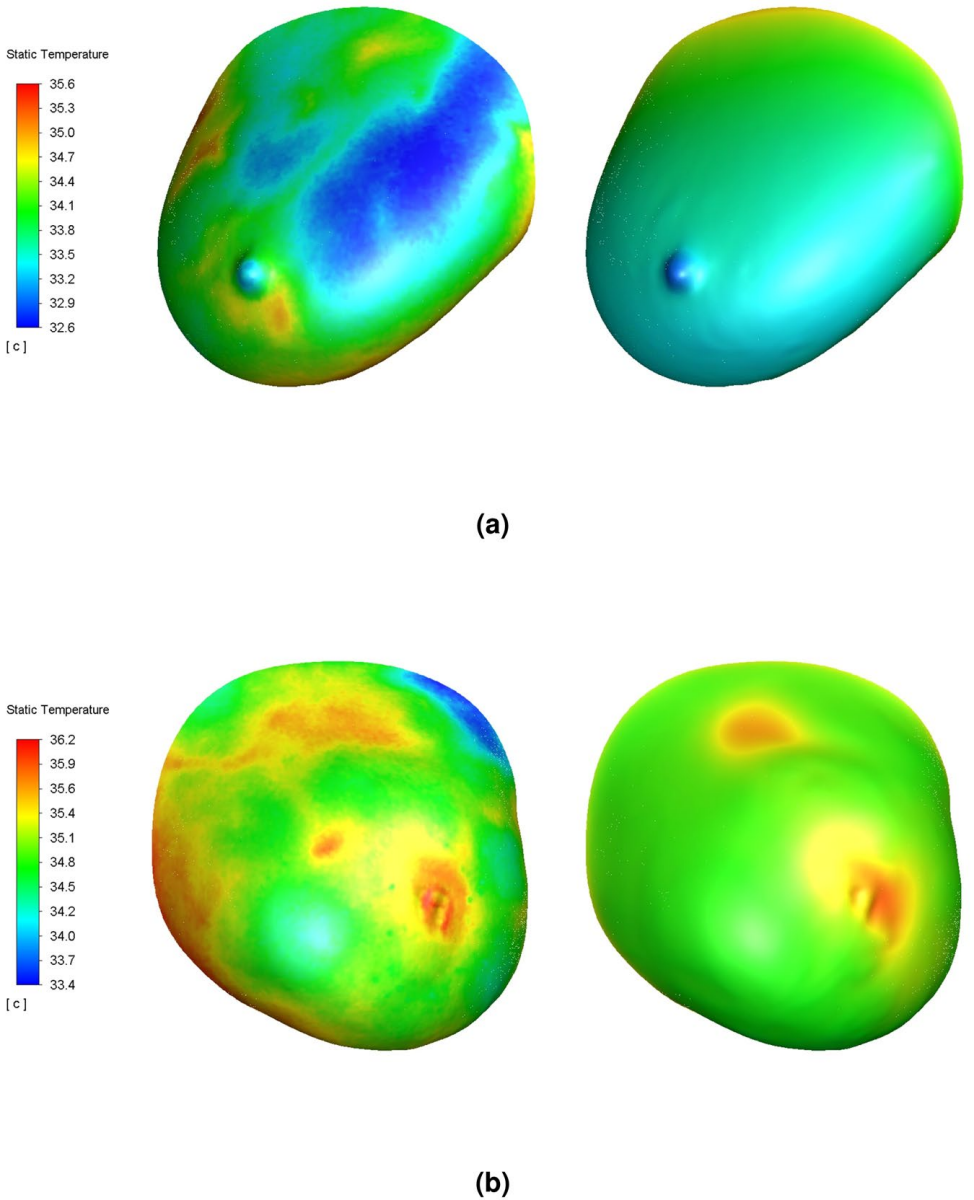
Surface temperature contours of a representative solution for the right normal breast model (**a**) and left malignant breast model (**b**). Subject’s frontal IR image shown on left for each breast model for reference; temperature map placement was accurate to within 5.0 mm. Solution for ${T}_{A}$ = 35.5°C and *h* = 5.0 W/m^2^-K case shown; all other (${\dot{Q}}_{m}^{{\rm{{\prime} }}{\rm{{\prime} }}{\rm{{\prime} }}},{\dot{\omega }}_{b}$) solutions reported herein were similar. For the right breast model, the mean surface temperature matched the IR image. For the left breast model, the two hotspots caused by the internal cancerous mass matched the IR image. The medial hotspot, which was due to a local blood vessel and not any underlying mass, was not recreated. Lighting shown to demonstrate 3D surfaces.

Solutions of (${\dot{Q}}_{m}^{{\rm{{\prime} }}{\rm{{\prime} }}{\rm{{\prime} }}},{\dot{\omega }}_{b}$) pairs were found for a range of arterial temperatures, ${T}_{A}$, and convective heat transfer coefficients, $h$ (or HTC). Results are presented in the following: Fig. 1 plots solutions for the right (Fig. 1a) and left (Fig. 1b) breast thermal models, which represent thermal characteristics of normal and cancerous breast tissues, respectively; each (${\dot{Q}}_{m}^{{\rm{{\prime} }}{\rm{{\prime} }}{\rm{{\prime} }}},{\dot{\omega }}_{b}$) solution pair is plotted along its corresponding ${T}_{A}$ line. For the left breast model, solutions are presented for each of the three tissues in the model (lower cancerous, upper cancerous, and non-cancerous tissues). Figure 2 demonstrates surface temperature contours for a representative solution from the right (Fig. 2a) and left (Fig. 2b) breast models, with the IR image shown for reference representing the true temperature distribution. Finally, Table 1 outlines all solutions found for each breast model.

**Table 1:** Solution pair values of (${\dot{Q}}_{m}^{{\rm{{\prime} }}{\rm{{\prime} }}{\rm{{\prime} }}},{\dot{\omega }}_{b}$) found for the subject’s right and left breast thermal models matching surface temperatures on IR images.

*T*_*A*_ [°C]	Ranges			Solutions								
	Breast model	${\dot{Q}}_{m}^{{\rm{{\prime} }}{\rm{{\prime} }}{\rm{{\prime} }}}$ [W/m^3^]	${\dot{\omega }}_{b}$ [s^−1^]	Breast tissue	5.0 W/m^2^-K		6.0 W/m^2^-K		7.0 W/m^2^-K		8.0 W/m^2^-K	
					${\dot{Q}}_{m}^{{\rm{{\prime} }}{\rm{{\prime} }}{\rm{{\prime} }}}$ [W/m^3^]	${\dot{\omega }}_{b}$ [s^−1^]	${\dot{Q}}_{m}^{{\rm{{\prime} }}{\rm{{\prime} }}{\rm{{\prime} }}}$ [W/m^3^]	${\dot{\omega }}_{b}$ [s^−1^]	${\dot{Q}}_{m}^{{\rm{{\prime} }}{\rm{{\prime} }}{\rm{{\prime} }}}$ [W/m^3^]	${\dot{\omega }}_{b}$ [s^−1^]	${\dot{Q}}_{m}^{{\rm{{\prime} }}{\rm{{\prime} }}{\rm{{\prime} }}}$ [W/m^3^]	${\dot{\omega }}_{b}$ [s^−1^]
35.5	Right	2.00E02–3.30E03	3.40E-04–1.75E-03	Normal	2.00E02	3.400E-04	7.20E02	5.765E-04	1.50E03	9.313E-04	2.50E03	1.386E-03
	Left	3.00E04–5.80E05	5.98E-03–1.63E-02	Non-cancerous	3.00E04	5.980E-03	3.90E04	6.152E-03	5.30E04	6.410E-03	6.70E04	6.668E-03
				Upper cancerous	1.03E05	7.356E-03	1.22E05	7.700E-03	1.49E05	8.216E-03	1.68E05	8.560E-03
				Lower cancerous	3.05E05	1.114E-02	3.78E05	1.252E-02	4.52E05	1.389E-02	5.25E05	1.527E-02
35.0	Right	2.00E02–6.80E03	3.40E-04–1.75E-03	Normal	1.30E03	5.750E-04	2.73E03	8.816E-04	4.40E03	1.237E-03	6.55E03	1.697E-03
	Left	3.00E04–7.00E05	5.98E-03–1.63E-02	Non-cancerous	5.20E04	6.324E-03	6.40E04	6.496E-03	8.00E04	6.754E-03	9.70E04	7.012E-03
				Upper cancerous	1.42E05	7.700E-03	1.64E05	8.044E-03	1.92E05	8.474E-03	2.09E05	8.732E-03
				Lower cancerous	3.99E05	1.166E-02	4.88E05	1.303E-02	5.66E05	1.424E-02	6.53E05	1.558E-02
34.5	Right	2.00E02–1.05E04	3.40E-04–1.75E-03	Normal	3.60E03	8.054E-04	5.65E03	1.086E-03	8.60E03	1.490E-03	NS	NS
	Left	3.00E04–8.20E05	5.98E-03–1.63E-02	Non-cancerous	8.30E04	6.668E-03	9.60E04	6.840E-03	1.16E05	7.098E-03	(1.35E05)	(7.356E-03)
				Upper cancerous	1.88E05	8.044E-03	2.01E05	8.216E-03	2.34E05	8.646E-03	(2.54E05)	(8.904E-03)
				Lower cancerous	5.04E05	1.217E-02	5.96E05	1.338E-02	6.88E05	1.458E-02	(7.83E05)	(1.582E-02)
34.0	Right	2.00E02–1.40E04	3.40E-04–1.75E-03	Normal	7.00E03	1.035E-03	1.00E04	1.341E-03	1.40E04	1.750E-03	NS	NS
	Left	3.00E04–9.40E05	5.98E-03–1.63E-02	Non-cancerous	1.21E05	7.012E-03	1.36E05	7.184E-03	1.51E05	7.356E-03	(1.74E05)	(7.614E-03)
				Upper cancerous	2.27E05	8.216E-03	2.42E05	8.388E-03	2.73E05	8.732E-03	(2.95E05)	(8.990E-03)
				Lower cancerous	6.06E05	1.252E-02	7.13E05	1.372E-02	8.10E05	1.482E-02	(9.16E05)	(1.602E-02)
33.5	Right	2.00E02–1.75E04	3.40E-04–1.75E-03	Normal	1.12E04	1.237E-03	1.56E04	1.595E-03	NS	NS	NS	NS
	Left	3.00E04–1.06E06	5.98E-03–1.63E-02	Non-cancerous	1.50E05	7.184E-03	1.67E05	7.356E-03	(1.93E05)	(7.614E-03)	(2.10E05)	(7.786E-03)
				Upper cancerous	2.70E05	8.388E-03	2.88E05	8.560E-03	(3.13E05)	(8.818E-03)	(3.39E05)	(9.076E-03)
				Lower cancerous	7.17E05	1.286E-02	8.23E05	1.393E-02	(9.26E05)	(1.496E-02)	(1.046E06)	(1.616E-02)
33.0	Right	2.00E02–2.13E04	3.40E-04–1.75E-03	Normal	1.63E04	1.416E-03	2.13E04	1.750E-03	NS	NS	NS	NS
	Left	3.00E04–1.18E06	5.98E-03–1.63E-02	Non-cancerous	1.83E05	7.356E-03	2.03E05	7.528E-03	(2.22E05)	(7.700E-03)	(2.41E05)	(7.872E-03)
				Upper cancerous	3.08E05	8.474E-03	3.27E05	8.646E-03	(3.56E05)	(8.904E-03)	(3.85E05)	(9.162E-03)
				Lower cancerous	8.35E05	1.320E-02	9.31E05	1.406E-02	(1.042E06)	(1.506E-02)	(1.180E06)	(1.630E-02)

Ranges (i.e., endpoints) per *T*_*A*_ from which (${\dot{Q}}_{m}^{{\rm{{\prime} }}{\rm{{\prime} }}{\rm{{\prime} }}},{\dot{\omega }}_{b}$) solutions were obtained shown for reference. NS entries indicate that no solution was found for the given (${\dot{Q}}_{m}^{{\rm{{\prime} }}{\rm{{\prime} }}{\rm{{\prime} }}},{\dot{\omega }}_{b}$) range. Solutions inside parentheses indicate that no corresponding solution was found in the right breast model. *T*_*A*_ = 36.0°C series not shown because no solutions were found for this series.

Regarding the right breast model, the accuracy of each solution was verified based on the calculated rate of convective heat loss from the anterior surface (based on the 33.8°C mean surface temperature over the entire breast); solutions were accurate to within 0.003 W of the theoretical value, which varied by HTC. The maximum internal temperature for all cases was 36.0°C. No solutions were found for HTC values greater than 8.0 W/m^2^-K given the (${\dot{Q}}_{m}^{{\rm{{\prime} }}{\rm{{\prime} }}{\rm{{\prime} }}},{\dot{\omega }}_{b}$) ranges selected for either of the two following reasons: First, the maximum internal breast temperature constraint was not satisfied (see Eq. 6), or second, insufficient internal heat was generated to overcome the convective surface cooling caused by the high HTC value. Similarly, no solutions were found for *T*_*A*_ = 36.0°C that satisfied the maximum internal temperature constraint, which was attributed to the posterior wall boundary condition (see Eq. 3).

Regarding the left breast model, solutions were found for all HTC and ${T}_{A}$ cases considered, contrary to the right breast model for the reason that no constraints were enforced in this model. Noteworthy were the large magnitudes of ${\dot{Q}}_{m}^{{\rm{{\prime} }}{\rm{{\prime} }}{\rm{{\prime} }}}$ in lower cancerous breast tissue relative to other tissues required to achieve sufficiently high surface temperatures at the nipple, particularly for lower ${T}_{A}$ values. This was attributed to the local region of skin thickening at the nipple. Maximum internal breast temperatures for each case varied between 42.2–50.8°C.

## Discussion

The solutions of normal and cancerous breast tissues presented herein represent a general range of possibilities regarding their thermal characteristics, depending on arterial temperature and environmental conditions (i.e., convective heat transfer coefficient and ambient temperature). For cancerous breast tissue specifically, it is important to note that solutions were subject-specific and cannot necessarily be applied to all breast cancers. However, the subject modeled herein represented a triple negative breast cancer and the thermal characteristics reported may be applicable to this molecular subtype of breast cancer in other subjects. In contrast, for normal breast tissue, solutions found for metabolic heat generation rate were generally less than 20,000 W/m^3^, which were comparable to the maximum value of 12,000 W/m^3^ reported by Gautherie^7^.

Mathematically, the Pennes temperature-dependent source term, ${(\rho c)}_{b}{\dot{\omega }}_{b}({T}_{A}-T)$, can represent either a heat source or heat sink, depending on whether the local tissue temperature is less than ${T}_{A}$ (heat source) or greater than ${T}_{A}$ (heat sink). This perfusion term balances the metabolic heat generation term, ${\dot{Q}}_{m}^{{\rm{{\prime} }}{\rm{{\prime} }}{\rm{{\prime} }}}$, in order to bring the local tissue temperature closer to ${T}_{A}$ according to the magnitude of ${\dot{\omega }}_{b}$. Physiologically, this term represents the thermoregulatory mechanism in perfused tissue, and without this term, unrealistically high tissue temperatures may be encountered. Accordingly, for the right breast model, any metabolic heat generation at *T*_*A*_ ≥ 36.0°C violated the maximum internal breast temperature constraint (see Eq. 6) and therefore yielded no solution, owing to the fact that a 36.0°C posterior wall temperature boundary condition was assigned based on measurements reported by Gautherie (see Eq. 3)^7^.

The slope of each ${T}_{A}$ series in Fig. 1 was unique to the (${\dot{Q}}_{m}^{{\rm{{\prime} }}{\rm{{\prime} }}{\rm{{\prime} }}},{\dot{\omega }}_{b}$) endpoints selected for that individual series (see Table 1). The ranges of (${\dot{Q}}_{m}^{{\rm{{\prime} }}{\rm{{\prime} }}{\rm{{\prime} }}},{\dot{\omega }}_{b}$) were determined based on preliminary trial-and-error modeling that yielded a solution under the most extreme condition modeled (here, *h* = 8.0 W/m^2^-K, *T*_*A*_ = 33.0°C). Various other (${\dot{Q}}_{m}^{{\rm{{\prime} }}{\rm{{\prime} }}{\rm{{\prime} }}},{\dot{\omega }}_{b}$) ranges per ${T}_{A}$ series were found to yield fewer solutions than the ranges assumed (not reported). Therefore, it was conceivable that an optimal (${\dot{Q}}_{m}^{{\rm{{\prime} }}{\rm{{\prime} }}{\rm{{\prime} }}},{\dot{\omega }}_{b}$) range existed that could yield even more solutions for greater HTC values than those reported herein. However, ascertaining an optimal (${\dot{Q}}_{m}^{{\rm{{\prime} }}{\rm{{\prime} }}{\rm{{\prime} }}},{\dot{\omega }}_{b}$) range would require a numerical parametric sensitivity study, which was beyond the scope of the present modeling study.

In the right (normal) breast thermal model, the following inter-variable relationships were observed: As ${T}_{A}$ decreased, not enough heat could be generated internally to produce a sufficiently high mean surface temperature of 33.8°C. That is, fewer solutions were found as ${T}_{A}$ decreased. This was attributed to the fact that the Pennes perfusion term (here, a heat sink), which is a function of ${T}_{A}$, counteracted any contribution from the ${\dot{Q}}_{m}^{{\rm{{\prime} }}{\rm{{\prime} }}{\rm{{\prime} }}}$ source term. The same observation was made as HTC increased for the same mathematical reasoning. Further, increasing ${\dot{Q}}_{m}^{{\rm{{\prime} }}{\rm{{\prime} }}{\rm{{\prime} }}}$ at a constant ${\dot{\omega }}_{b}$ yielded higher surface temperatures, which varied by case. Conversely, increasing ${\dot{\omega }}_{b}$ at a constant ${\dot{Q}}_{m}^{{\rm{{\prime} }}{\rm{{\prime} }}{\rm{{\prime} }}}$ brought tissue temperatures closer to ${T}_{A}$ according to the magnitude of ${\dot{\omega }}_{b}$.

**Figure 3:**
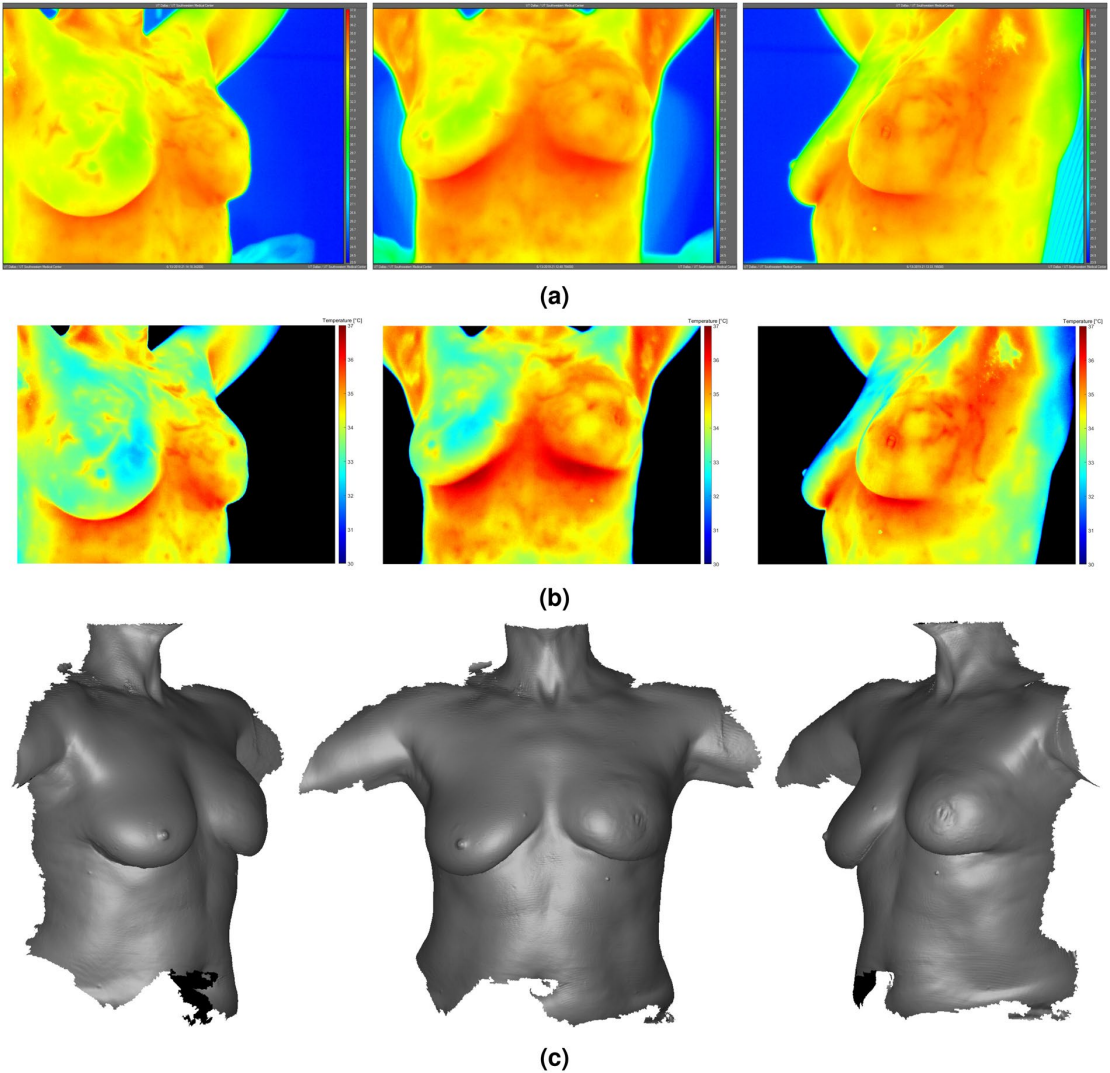
Infrared images (**a**,**b**) and 3D breast surface scans (**c**) of Subject 03. IR images in (**a**) were exported directly from FLIR ResearchIR Max; IR images in (**b**) were processed using MATLAB to modify background color to black. Clear thermovascular asymmetry was observed between breasts due to generalized hyperthermia in subject’s left breast (see Table 2 for summary of temperatures). Physical deformity in subject’s left breast due to malignancy was evident at presentation during research procedures. High-resolution IR images available for download online (see Supplementary Figs. S1–S6).

Interestingly, in the left (cancerous) breast thermal model, the elevated perfusion rates characteristic of cancerous tissue were required for all tissues—including non-cancerous breast tissue—in order to find solutions. That is, normal perfusion rates did not suffice in raising the mean surface temperature (excluding hotspots) to the 34.9°C metric. This observation was attributed to the fact that normal perfusion rates, which are approximately ten times lower than cancerous tissue, limited the magnitude of the Pennes perfusion term (here, a heat source). This finding indicated that for invasive and aggressive breast cancers (as observed in the subject), the entire breast may exhibit elevated blood perfusion rates characteristic of cancerous tissue, not only the malignant tumor itself. This finding was reasonable given the 1.2°C mean temperature difference between breasts and generalized hyperthermia on the subject’s left breast based on the subject’s IR images (see Fig. 3a,b and Table 2).

**Table 2:** Summary of temperatures based on Subject 03’s frontal-view IR image.

Subject 03: IR image temperature summary [°C]			
	Max	Mean	Min
Left breast (cancerous)	36.2	35.0	33.4
Right breast (normal)	35.6	33.8	32.6
Difference	0.6	1.2	0.8

Values determined in FLIR ResearchIR Max by drawing bounded regions around each breast.

It was also observed in the left breast thermal model that given the physiologic modeling assumptions made, the ${\dot{Q}}_{m}^{{\rm{{\prime} }}{\rm{{\prime} }}{\rm{{\prime} }}}$ ranges of cancerous tissue reported by Gautherie did not produce sufficiently high surface temperatures to meet the hotspot target metrics for the subject modeled in this study. In fact, based on the 10 cm tumor size exhibited by the subject (with an unknown doubling time), the appropriate metabolic heat generation rate per Gautherie’s relation was unclear^7^. Also, it is impractical to clinically ascertain tumor doubling times with routine diagnostic imaging exams. These two attributes (large tumor size, unknown doubling time) reasonably justified not implementing Gautherie’s ${\dot{Q}}_{m}^{{\rm{{\prime} }}{\rm{{\prime} }}{\rm{{\prime} }}}$ relation into the thermal model. Although results presented herein do not necessarily disprove Gautherie’s range of ${\dot{Q}}_{m}^{{\rm{{\prime} }}{\rm{{\prime} }}{\rm{{\prime} }}}$, they suggest that other ranges may exist based on various breast cancer factors including histology and molecular subtype. Accordingly, this work is distinguished from Recinella *et al*.’s recent notable work in that the primary thermal modeling parameters (blood perfusion rate and metabolic heat generation rate of cancer) were herein modeled as adjustable variables rather than incorporating Gautherie’s ${\dot{Q}}_{m}^{{\rm{{\prime} }}{\rm{{\prime} }}{\rm{{\prime} }}}$ relation^46^.

In this study, the subject’s medial hotspot was not modeled for the reason that it was attributed to a local blood vessel and not to the underlying malignant tumor. Accurately recreating the subject’s medial hotspot would require incorporating the internal vasculature (i.e., blood vessel branching structure) into the thermal model. Therefore, these results highlight the inherent limitation to implementing Pennes’ bioheat equation. Modeling the computational fluid dynamics (CFD) of the detailed 3D vasculature reconstructed from MR imaging—with accurate hemodynamic velocities and arterial temperatures—has recently made progress and is needed to achieve a higher degree of fidelity in the thermal modeling of any tissue^47,48^.

The following were three limitations to the modeling study presented herein. First, as previously mentioned, modeling results were subject-specific and are not necessarily applicable to all breast cancers. Second, the metabolic heat generation rate of skin was unknown. No empirical data were found in the literature, and in an effort to avoid modeling arbitrary values, it was consequently assumed to be zero. Skin tissue likely has a degree of metabolic heat generation, even if it were to have a minimal impact on surface temperatures. Accordingly, the thermal characteristics of breast cancer reported herein may be interpreted as upper-limit values, pending empirical data on skin’s metabolic heat generation rate. Third, high maximum internal temperatures were observed for lower cancerous tissue in the left breast model, despite the reasonable matching of surface temperatures on the breast. In fact, internal temperatures in cancerous tissue exceeding 40°C approach hyperthermia treatment ranges^49–52^. It is expected that incorporating the detailed internal vasculature would resolve this limitation and eliminate the need for Pennes’ bioheat equation altogether.

In summary, the following are key takeaways from the thermal modeling results presented herein:

1. Modeling results matched surface temperatures from the subject’s IR images reasonably well.
2. The high (${\dot{Q}}_{m}^{{\rm{{\prime} }}{\rm{{\prime} }}{\rm{{\prime} }}},{\dot{\omega }}_{b}$) values for the subject reported herein may be characteristic of triple negative breast cancer cases at late stages with large tumor sizes and may be indicative of how high heat-producing these aggressive cancers are.
3. For subjects exhibiting a generalized hyperthermia, the entire breast may exhibit elevated, cancer-level blood perfusion rates, not only the malignant tumor itself.
4. Gautherie’s reported metabolic heat generation rates were insufficient in producing large enough surface temperatures for this particular subject^7^.

Potential clinical applications for the computational modeling of breast cancer include: Monitoring tumor response to cancer treatment over other imaging modalities; tracking tumor growth over time, including from presentation to surgical removal^53^; and hyperthermia cancer treatment^49–52^.

In conclusion, the present study developed a computational thermal model of breast cancer based on high-resolution IR images, real 3D breast geometries, and real tumor definition from a breast cancer subject. The novelty of the study was its use of clinical data to construct and calibrate the model. The goal of the study was to quantify the thermal characteristics of breast cancer. The results reported herein represent a first step toward the accurate computational thermal modeling of breast cancer. Clearly, the female breast is a complex, inhomogeneous tissue. Thus, results provide estimates of the ranges expected regarding the metabolic heat generation rate of breast cancer, particularly for the aggressive triple negative subtype exhibited by the subject. Future directions of this study include validating the calibrated model with future female subjects presenting with similar breast cancer characteristics (here, molecular subtype, stage, and tumor size) and incorporating the real 3D breast vasculature into the model as discussed earlier^47,48^.

## Methods

The present study was comprised of a clinical study and computational modeling effort. Clinical data were used to develop the thermal model. Specifically, 3D surface scans defined the breast geometry; diagnostic imaging procedures defined the tumor geometry; and IR images were used to calibrate the model.

### Clinical study

The clinical study underwent review and approval by the following institutions: The University of Texas Southwestern Medical Center’s Institutional Review Board (IRB Study No. STU-2018-0370), Breast Cancer Disease Oriented Team (DOT), and Protocol Review and Monitoring Committee (PRMC); The University of Texas at Dallas’ Institutional Review Board (IRB Study No. 19–46); and finally Parkland Health & Hospital System’s Office of Research Administration (Study No. 27044). The clinical study commenced after receiving final approval from all institutions. All research procedures were performed in accordance with relevant guidelines and regulations. Eleven female subjects who were referred for breast biopsy on the basis of abnormal radiologic findings were enrolled in the study after obtaining written informed consent. Clinical data were collected between 06/13/2019–09/26/2019. Clinical assessments considering all subjects will be outlined in a future communication.

#### Procedures and equipment

Subjects entered and sat in the examination room and disrobed above the waist. Subjects had previously been thermally acclimated to the environment. High-resolution IR images were recorded with a FLIR A655sc infrared camera (FLIR Systems Inc., Wilsonville, OR, USA). The IR camera had a 640 × 480 resolution and 30 mK (0.030°C) sensitivity. The standard lens on the IR camera was used (25° field of view, 24.6 mm focal length) and subjects were positioned approximately 1 m away from the IR camera. IR images were processed using FLIR ResearchIR Max 4.40.8.28, a vendor-provided proprietary software. The emissivity was set to a constant value of 0.98 as has been previously determined empirically for human skin^54–56^. Additionally, IR images were post-processed using MATLAB 2019b (The MathWorks Inc., Natick, MA, USA).

Then, 3D breast surface scans were recorded with an Artec Eva Lite (Artec Europe SARL, Luxembourg), a portable, handheld 3D scanner with an accuracy of 100 μm and resolution of 500 μm. The 3D scanner was held approximately 50–60 cm away from subjects’ breasts and gently swept in an airbrush motion for approximately 30–60 seconds to fully record the 3D topography of both breasts. 3D scans were processed using Artec Studio 13 Professional, a vendor-provided proprietary software.

Finally, the ambient temperature in the clinical examination room ranged between 22.0–24.0°C. Ambient temperature was measured with an Omega HH506RA digital thermometer (Omega Engineering Inc., Norwalk, CT). The thermometer had a 0.1°C resolution and ±0.3°C accuracy. A T-type thermocouple with 36 AWG wire (0.13 mm diameter) with an exposed junction was used with the digital thermometer (Omega 5SRTC-TT-T-36-36). The thermocouple had a ±0.5°C accuracy (“special limits of error” grade wire). Manufacturer datasheets for all equipment are publicly available online.

#### Clinical data for representative subject

Clinical data and modeling results are herein presented for a representative subject, Subject 03. Subject 03 was selected from among the study population due to the subject’s clear thermal asymmetry between breasts, visible physical deformity, and aggressive breast cancer subtype. Subject 03 (herein referred to as “the subject”) was histologically diagnosed with Stage 4 breast cancer in the left breast (invasive ductal carcinoma of no special type, Grade 2, triple negative receptor status).

The subject exhibited a mean thermal asymmetry between breasts of 1.2°C. In addition to the generalized hyperthermia, the subject’s left breast exhibited three localized (or focal) regions of increased temperatures: (1) the nipple region (herein, “nipple hotspot”); (2) approximately 8.0 cm superior to the nipple at 11 o’clock (herein, “superior hotspot”); and (3) approximately 5.0 cm medial to the nipple at 10 o’clock (herein, “medial hotspot”). The nipple and superior hotspots were caused by the underlying malignant lesion (i.e., tumor or mass); that is, the cancer was located directly behind these two regions. The medial hotspot, in contrast, was not due to any underlying mass, but rather was attributed to a local blood vessel. The subject’s IR images are presented in Fig. 3a,b and are summarized in Table 2. The subject’s two hotspots that were due to the underlying cancerous mass were the focus of the modeling effort.

**Figure 4:**
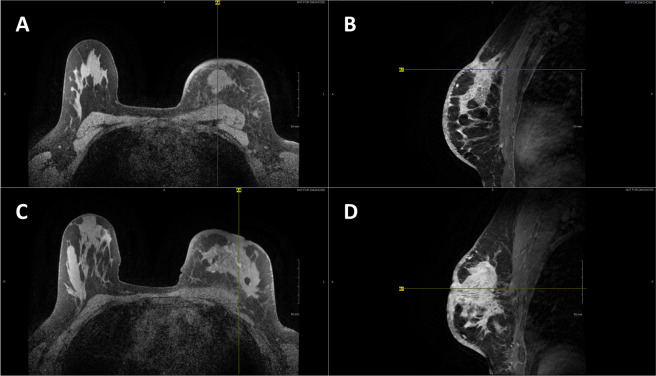
Axial (**A**) and sagittal (**B**) T1 contrast-enhanced MR images of Subject 03’s superior hotspot. Axial (**C**) and sagittal (**D**) T1 contrast-enhanced MR images of Subject 03’s nipple hotspot. Localized regions of skin retraction and skin thickening evident from MR images.

The subject’s left breast also exhibited an overall skin thickening with an average thickness of approximately 5.0 mm based on MR images (in contrast to the right breast with an average skin thickness of 1.5 mm). The subject’s left breast also exhibited two small regions of skin retraction corresponding to two hotspots observed: Nipple region and 8.0 cm superior to the nipple. The two regions of skin retraction were visible in 3D scans. The subject’s 3D scans and MR images are presented in Figs. 3c and 4A–D, respectively.

### Thermal model

The computational thermal model of breast cancer was constructed using the ANSYS Workbench 2019 R2 version 19.4 suite (ANSYS Inc., Canonsburg, Pennsylvania, USA), which used ANSYS Meshing and ANSYS Fluent. ANSYS Meshing was used to generate the mesh for the geometry and ANSYS Fluent was used to define the governing equation, assign thermal properties, apply boundary conditions, and numerically solve the governing equation. ANSYS Fluent implements the finite volume method (FVM) to integrate the governing conservation laws over each control volume cell in the geometric domain and numerically solve the resulting discretized equations.

#### Governing equation and boundary conditions

Absent of the subject’s real internal vasculature definition, Pennes’ bioheat equation was solved as the governing equation in order to obtain the surface temperature distribution of the breast (see Eq. 1). Here, $T$ represents the temperature of the tissue, $\rho$ represents its density, $c$ represents its specific heat, $k$ represents its thermal conductivity, ${\dot{\omega }}_{b}$ represents its blood perfusion rate, ${T}_{A}$ represents the temperature of its arterial blood supply, and ${\dot{Q}}_{m}^{{\rm{{\prime} }}{\rm{{\prime} }}{\rm{{\prime} }}}$ represents its metabolic heat generation rate. Pennes’ bioheat equation is a modified form of the heat equation that includes an additional temperature-dependent source term, ${(\rho c)}_{b}{\dot{\omega }}_{b}({T}_{A}-T)$. The justification for using Pennes’ bioheat equation as an analysis tool, despite its physiologic assumptions and limitations, has been previously established^57–60^.

1 $$\rho c\frac{{\rm{\partial }}T}{{\rm{\partial }}t}={\rm{\nabla }}\cdot k{\rm{\nabla }}T+{(\rho c)}_{b}{\dot{\omega }}_{b}({T}_{A}-T)+{\dot{Q}}_{m}^{{\rm{{\prime} }}{\rm{{\prime} }}{\rm{{\prime} }}}$$

In order to solve this governing equation using ANSYS Fluent, a user-defined function (UDF) that enforced the Pennes temperature-dependent perfusion source term in the governing equation was required. The UDF is a short code in the C++ programming language using the appropriate Fluent macros; in this case, the “DEFINE_SOURCE” macro was used. The UDF was written and loaded into the model.

The numerical validity of the custom UDF was confirmed by solving a simple, *ad hoc* hemispherical model using both ANSYS Fluent 2019 R2 and COMSOL Multiphysics 5.4 and comparing results. COMSOL Multiphysics contains a built-in bioheat transfer module that applies the temperature-dependent source term to the numerical solution. By applying the same mesh parameters, material properties, boundary conditions, thermal characteristics, and numerical solution settings in both software, the steady-state models were observed to be identical to within 0.01°C with regard to the maximum and minimum temperature values and internal and surface temperature distributions; results were omitted for brevity. This exercise validated the accuracy of the UDF written for ANSYS Fluent.

Because the steady-state model did not simulate any fluid flow, the ANSYS Fluent flow solver was turned off. The energy convergence criterion was set to 1E-12, which was sufficient for a numerically accurate solution. The energy under-relaxation factor, which controls the change in temperature over each iteration, was set to 0.95 in order to avoid divergence. Solutions generally required 20 iterations to satisfy this convergence criterion using the parallel processor solver setting on a quad-core processor computer. Results were verified to vary by less than 0.001°C with a convergence criterion of 1E-15.

Finally, the boundary conditions selected were consistent with prior modeling studies. First, a variable convective heat transfer coefficient was assigned to the anterior surface area of the breast ($n$ normal direction at any point on the surface; see Eq. 2). A reference ambient temperature (${T}_{\infty }$) of 23.0°C was assigned, based on the room temperature measured during the IR imaging procedure. Second, the temperature of the surface posterior to breast tissue (“posterior wall”) was held at a constant and uniform temperature of ${T}_{P}$ = 36.0°C (i.e, isothermal), based on *in vivo* temperature measurements of deep breast tissue reported by Gautherie (see Eq. 3)^7^. This posterior wall represented the anterior surface of the pectoral muscle on the thoracic wall and was constructed based on the subject’s MR imaging. Additionally, the connecting surfaces between the anterior and posterior surfaces were assumed adiabatic (see Eq. 4).

2 $$-k\frac{\partial T}{\partial n}=h({T}_{s}-{T}_{\infty })\,({\rm{anterior}}\,{\rm{surface}}\,{\rm{only}})$$

3 $${T|}_{x,y,z=0}\,={T}_{P}\,({\rm{posterior}}\,{\rm{surface}}\,{\rm{only}})$$

4 $$\frac{\partial T}{\partial n}=0\,({\rm{connecting}}\,{\rm{surfaces}}\,{\rm{only}})$$

#### Geometry

Real 3D breast surface geometries as recorded by the 3D scanner were imported into the thermal model (see Fig. 3c). This allowed for an accurate representation of the subject’s unique breast shape (i.e., the natural ptosis of the breast) and convective surface area.

With regard to modeling workflow, Artec Studio 13 Professional exported triangular mesh files (STL format) from raw 3D scan data. STL mesh files were then imported into Solidworks 2018 (Dassault Systemes, Velizy-Villacoublay Cedex, France), a computer-aided design (CAD) software, to generate a smooth, continuous surface using the “ScanTo3D” add-in. Then, using standard functions in Solidworks, a solid body was generated from the surface. Finally, the solid body CAD model was imported into ANSYS Meshing for discretization and later into ANSYS Fluent for numerical solution.

**Figure 5:**
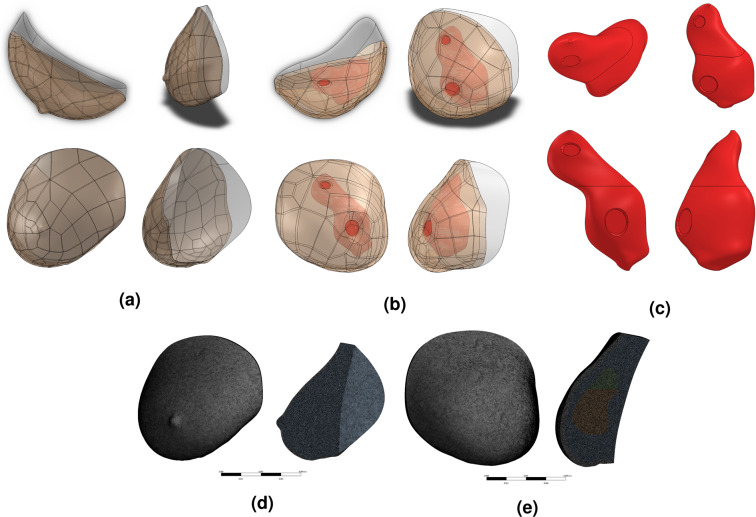
Standard orthographic views of Subject 03’s right breast (**a**), left breast (**b**), and tumor (**c**) CAD models constructed in Solidworks illustrating external and internal geometries. Frontal and cross-sectional views of Subject 03’s right (**d**) and left (**e**) breast thermal models illustrating meshes applied for computational solutions. Tumor geometry approximated from MR imaging data and included regions of skin retraction and skin thickening. Close inspection of the tumor geometry reveals the lower and upper portions, each modeled with varying metabolic and vascular properties. Anterior breast surfaces defined by 3D scans (see Fig. 3c).

A separate CAD model was constructed for each of the subject’s breasts (see Fig. 5a–c). Each breast CAD model consisted of a skin layer on the anterior surface, breast tissue, and cancerous tissue (if applicable) representing the tumor. The posterior surface boundary in each model was created based on the natural curvature of the pectoral muscle’s anterior surface according to corresponding MR images.

Skin layers were modeled based on MR images. The subject’s right breast was modeled with a uniform skin thickness of 1.5 mm. The subject’s left breast, however, was modeled with a uniform skin thickness of 5.0 mm that locally thickened to 10.0 mm (nipple region) and 6.5 mm (superior to nipple) as measured on MR images. Similarly, the location and irregular geometry of the internal tumor were approximated based on multiple MR image cross-sections (see Fig. 5b).

Radii from the posterior surface to the anterior surface of each CAD model were approximately 5.5 cm (left breast) and 5.3 cm (right breast). Bounding box dimensions (i.e., geometric envelope) of each model, in transverse × axial × depth format, were approximately 16.3 × 15.4 × 9.5 cm (left breast) and 16.0 × 14.1 × 8.7 cm (right breast). Solid volumes for each breast model were approximately 876.1 cm^3^ (left breast) and 588.2 cm^3^ (right breast); the discrepancy in volumes was explained by the deformity in the left breast caused by malignancy (see Fig. 3c). Exterior convective surface areas were approximately 312.0 cm^2^ (left breast) and 267.5 cm^2^ (right breast), derived from 3D surface scans.

Due to the irregular geometry of each breast, 1.0 mm tetrahedral mesh elements (maximum element size) were applied in ANSYS Meshing, which were later converted into polyhedral elements in ANSYS Fluent for faster computational processing. Results between tetrahedral and polyhedral mesh types were verified to be consistent to less than 0.005°C, which was approximately 10 times less than the IR camera’s sensitivity. Final polyhedral mesh sizes ranged between 5.1–7.7 million nodes, 6.1–9.1 million faces, and 0.9–1.3 million cells, depending on breast model (see Fig. 5d,e). Solutions reported herein were verified to be independent of mesh size; temperatures varied by less than 0.004°C when mesh size was reduced to 0.7 mm and 0.5 mm tetrahedrons.

#### Thermophysical properties

Thermophysical properties reported in the literature based on empirical data were assumed in the model as outlined in Table 3. Each tissue was modeled with homogeneous properties, with the exception of cancerous tissue as discussed in the proceeding section. Due to the subject’s heterogeneously dense classification of breast tissue based on mammograms (not shown), glandular breast tissue properties were assigned. Blood perfusion rates of cancerous breast tissue reported in the literature were approximately ten times greater than of normal breast tissue^61,62^.

**Table 3:** Summary of thermophysical properties assumed in the thermal model, derived from empirical measurements reported in the literature.

Tissue	*k* [W/m-K]	*ρ* [kg/m^3^]	*c* [J/kg-K]	${\dot{\omega }}_{b}$ [s^−1^]	${\dot{Q}}_{m}^{{\rm{{\prime} }}{\rm{{\prime} }}{\rm{{\prime} }}}$ [W/m^3^]
Skin	0.266 (^69^ from^70^)	1,110 (^69^)	3,230 (^71^)	2.22E-3 (^69^ from^72^)	—
Breast, normal	0.322 (^7^)	1,020 (^69^)	3,060 (^71^)	Variable: 3.40E-4–1.75E-3 (^62^)	Variable
Breast, cancerous	0.564 (^69^ from^73^)	1,020 (^69^)	3,060 (^71^)	Variable: 5.98E-3–1.63E-2 (^62^)	Variable
Blood	—	1,060 (^69^)	3,840 (^69^)	—	—

*In vitro* values selected when available in order to separate perfusion effects. Blood perfusion rates converted from units of [mL/min-g] to [s^−1^] using the corresponding solid tissue density. Density and specific heat values shown for reference but not used in the steady-state model.

The arterial blood temperatures of the skin, breast, and cancerous tissues in the model (${T}_{A,S}$, ${T}_{A,B}$, and ${T}_{A,C}$, respectively) were considered variable and assumed to be equal (see Eq. 5) based on the physiologic assumption that the same blood temperature supplied all three tissues. Arterial blood temperatures ranging between 33.0–35.5°C were considered, based on temperature measurements of the internal mammary artery reported in the literature^7,63,64^. Arterial temperatures, along with the thermophysical properties of blood outlined in Table 3, were incorporated into the UDF.

5 $${T}_{A,S}={T}_{A,B}={T}_{A,C}$$

Finally, the perfusion rate of skin was considered constant based on values reported in the literature (see Table 3). The metabolic heat generation of skin was neglected due to its low volume relative to the underlying breast tissue and the lack of data available in the literature.

#### Parametric formulation and target metrics for model calibration

In this study, ${\dot{Q}}_{m}^{{\rm{{\prime} }}{\rm{{\prime} }}{\rm{{\prime} }}}$ and ${\dot{\omega }}_{b}$ of normal and cancerous breast tissues were modeled as adjustable variables in order to determine the (${\dot{Q}}_{m}^{{\rm{{\prime} }}{\rm{{\prime} }}{\rm{{\prime} }}},{\dot{\omega }}_{b}$) solution pairs that recreated the surface temperatures observed on the subject’s IR images. The subject’s right breast was used to determine the thermal characteristics of normal breast tissue in the right normal breast model (see Fig. 5a), whereas the subject’s left breast was used to determine the thermal characteristics of cancerous and non-cancerous breast tissues in the left cancerous breast model (see Fig. 5b). The ranges of adjustable variables assumed per tissue in each model are outlined in Table 1. In contrast, the properties of skin tissue in each model were kept constant for all solutions (see Table 3).

For each breast model, the ranges of blood perfusion rate were constrained to values reported by Delille *et al*. for normal and cancerous breast tissues as outlined in Table 3^62^. Each ${\dot{\omega }}_{b}$ range was held constant for all ${T}_{A}$ and HTC cases simulated. Next, a corresponding range of metabolic heat generation rate was assigned to each breast tissue, which varied by ${T}_{A}$ case. A linearly proportional relationship was assumed between ${\dot{Q}}_{m}^{{\rm{{\prime} }}{\rm{{\prime} }}{\rm{{\prime} }}}$ and ${\dot{\omega }}_{b}$; that is, a low ${\dot{Q}}_{m}^{{\rm{{\prime} }}{\rm{{\prime} }}{\rm{{\prime} }}}$ corresponded to a low ${\dot{\omega }}_{b}$ and a high ${\dot{Q}}_{m}^{{\rm{{\prime} }}{\rm{{\prime} }}{\rm{{\prime} }}}$ corresponded to a high ${\dot{\omega }}_{b}$. This physiologic modeling assumption was supported by recent blood oxygen level-dependent (BOLD) resting-state functional magnetic resonance imaging (rs-fMRI) observations regarding the increased metabolic and vascular activity in brain tumors^65,66^. Solutions for each tissue were found within the (${\dot{Q}}_{m}^{{\rm{{\prime} }}{\rm{{\prime} }}{\rm{{\prime} }}},{\dot{\omega }}_{b}$) ranges selected for each ${T}_{A}$ series (i.e., solutions lied along the solid ${T}_{A}$ lines illustrated in Fig. 1). The ${\dot{Q}}_{m}^{{\rm{{\prime} }}{\rm{{\prime} }}{\rm{{\prime} }}}$ relation reported by Gautherie was not used in order to ensure the necessary degree of freedom in finding solutions.

Moreover, because breast tissue under normal physiologic conditions cannot physically exhibit higher temperatures than the posterior wall, a constraint on the maximum internal breast temperature was enforced for the right breast model (see Eq. 6). This constraint ensured the exclusion of extraneous solutions yielding artificially high internal temperatures for the right normal breast. This constraint was not enforced in the cancerous breast model due to the underlying thermophysiologic assumption that cancerous tissue (i.e., tumor) represented an internal heat source for the subject.

6 $${T}_{B,{\rm{\max }}}\le {T}_{P}\,({\rm{constraint}}:\,{\rm{right}}\,{\rm{breast}}\,{\rm{only}})$$

Finally, owing to the non-homogeneous vascularization possible within a tumor^67,68^, the tumor was modeled with two regions (upper and lower portions) of varying metabolic heat generation (and proportional blood perfusion) in order to best match clinical data. Figure 5c demonstrates the upper and lower portions of the cancerous tissue, which was partitioned at the approximate axial midpoint between the two regions of skin retraction outlined earlier. Thermal characteristics of the lower cancerous tissue governed the subject’s nipple hotspot, whereas the upper cancerous tissue governed the subject’s superior hotspot. This heterogeneity in tumor metabolic heat generation and perfusion provided an additional degree of freedom in finding solutions.

Therefore, in all, solution pairs of (${\dot{Q}}_{m}^{{\rm{{\prime} }}{\rm{{\prime} }}{\rm{{\prime} }}},{\dot{\omega }}_{b}$) were determined for a total of four tissues: Normal breast tissue (right breast model), non-cancerous breast tissue (left breast model), upper cancerous breast tissue (left breast model), and lower cancerous breast tissue (left breast model). Solution pairs were found for various HTC cases by marching along each ${T}_{A}$ line, solving the model with the specified parameters, analyzing resulting surface temperatures, and comparing with the subject’s IR images. For the right normal breast model, the mean surface temperature over the entire breast (33.8°C) was the sole metric for finding solutions, given its constraint (see Eq. 6). Here, (${\dot{Q}}_{m}^{{\rm{{\prime} }}{\rm{{\prime} }}{\rm{{\prime} }}},{\dot{\omega }}_{b}$) solutions of normal breast tissue governed the mean surface temperature. In contrast, for the left malignant breast, three metrics were used to find solutions: The mean temperatures of the two hotspots caused by the underlying malignant mass (nipple hotspot 35.6°C and superior hotspot 35.5°C) and the mean temperature of surrounding skin excluding hotspots (34.9°C). Here, (${\dot{Q}}_{m}^{{\rm{{\prime} }}{\rm{{\prime} }}{\rm{{\prime} }}},{\dot{\omega }}_{b}$) solutions of lower cancerous breast tissue governed the nipple hotspot, (${\dot{Q}}_{m}^{{\rm{{\prime} }}{\rm{{\prime} }}{\rm{{\prime} }}},{\dot{\omega }}_{b}$) solutions of upper cancerous breast tissue governed the superior hotspot, and (${\dot{Q}}_{m}^{{\rm{{\prime} }}{\rm{{\prime} }}{\rm{{\prime} }}},{\dot{\omega }}_{b}$) solutions of non-cancerous breast tissue governed the surrounding surface temperatures. The medial hotspot, attributed to a local blood vessel and not any internal cancerous tissue, was not modeled.

## Supplementary information

Supplementary Information.

Supplementary Information2.

Supplementary Information3.

Supplementary Information4.

Supplementary Information5.

Supplementary Information6.

Supplementary Information7.
