# Supplementary material for: Determining the thermal characteristics of breast cancer based on high-resolution infrared imaging, 3D breast scans, and magnetic resonance imaging
Source: Sci Rep. 2020 Jun 22;10:10105. doi: 10.1038/s41598-020-66926-6 (PMC7308290; doi:10.1038/s41598-020-66926-6)
Supplement: Supplementary file 1 — Supplementary Information. [file 41598_2020_66926_MOESM1_ESM.pdf]

Determining the thermal characteristics of breast cancer based on high-resolution infrared imaging, 3D breast scans, and magnetic resonance imaging

Adolfo Lozano III[1,2], Jody C. Hayes[3], Lindsay M. Compton[3], Jamasp Azarnoosh[1], and Fatemeh Hassanipour[1,*]

[1]Department of Mechanical Engineering, The University of Texas at Dallas, 800 W Campbell Rd, Mailstop EC-38, Richardson, TX 75080, USA
[2]Raytheon Space and Airborne Systems, 13510 North Central Expressway, Mailstop 212, Dallas, TX 75243, USA
[3]Department of Radiology, The University of Texas Southwestern Medical Center, 5323 Harry Hines Blvd., Dallas, TX 75390, USA
[*]fatemeh@utdallas.edu

Supplementary Information

Supplementary Figure S1: Subject 03’s right-view static infrared image (exported from FLIR ResearchIR Max).

Supplementary Figure S2: Subject 03’s frontal-view static infrared image (exported from FLIR ResearchIR Max).

Supplementary Figure S3: Subject 03’s left-view static infrared image (exported from FLIR ResearchIR Max).

Supplementary Figure S4: Subject 03’s right-view static infrared image (processed in Mathworks MATLAB).

Supplementary Figure S5: Subject 03’s frontal-view static infrared image (processed in Mathworks MATLAB).

Supplementary Figure S6: Subject 03’s left-view static infrared image (processed in Mathworks MATLAB).
