# Supplementary figures and images for: Determining the thermal characteristics of breast cancer based on high-resolution infrared imaging, 3D breast scans, and magnetic resonance imaging
Source: Sci Rep. 2020 Jun 22;10:10105. doi: 10.1038/s41598-020-66926-6 (PMC7308290; doi:10.1038/s41598-020-66926-6)

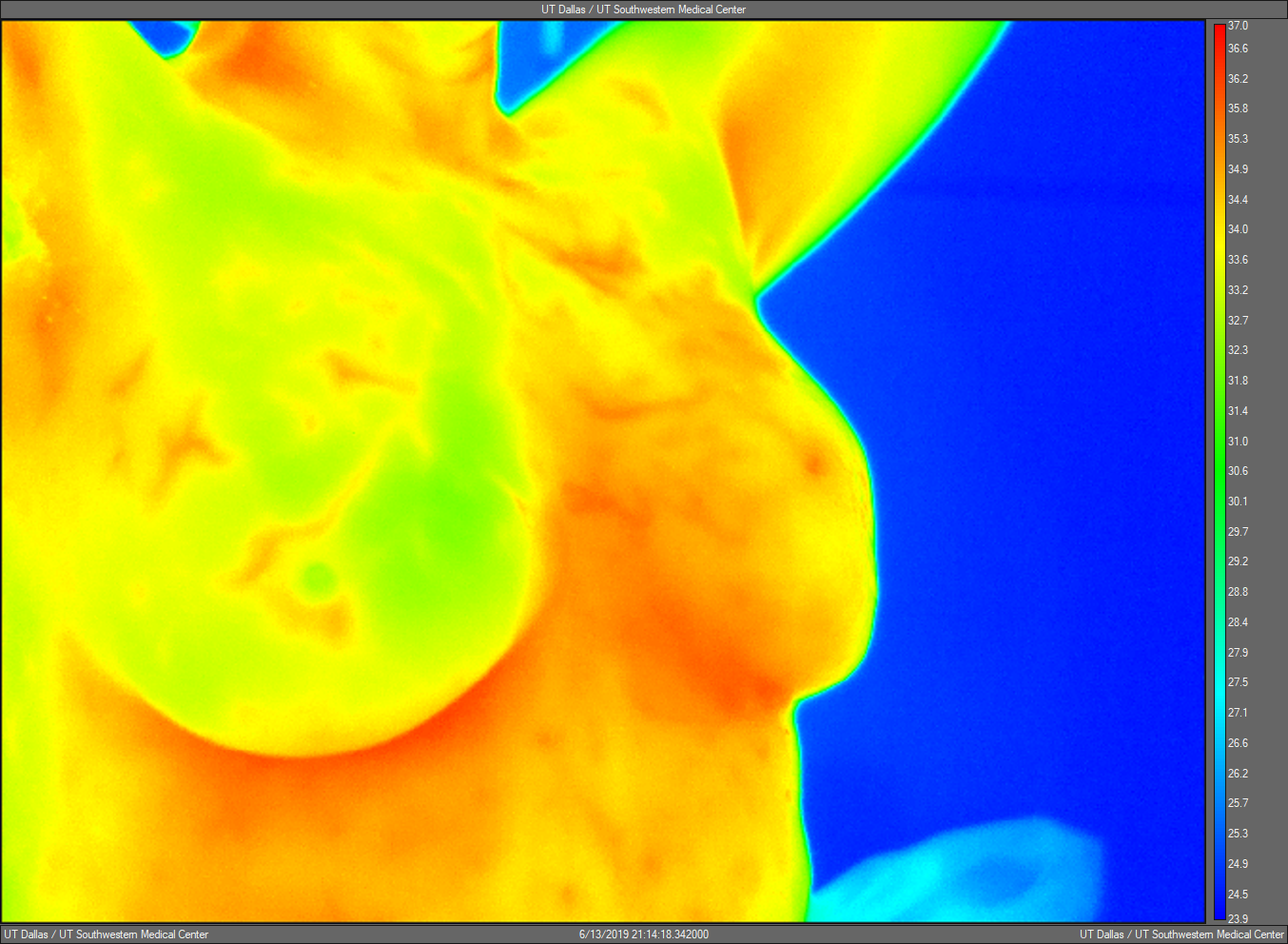

Supplement: Supplementary file 2 — Supplementary Information2. [file 41598_2020_66926_MOESM2_ESM.tif]

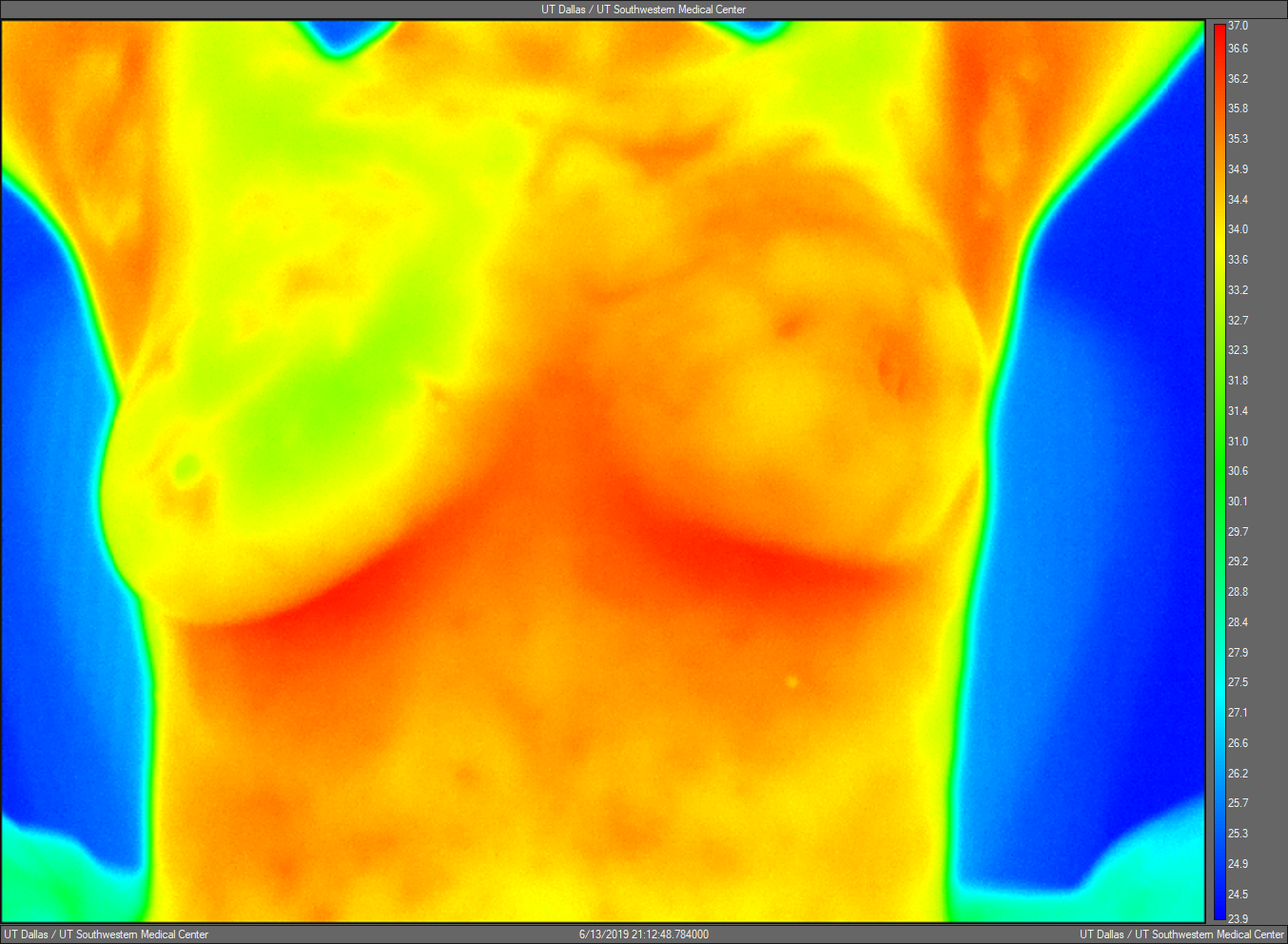

Supplement: Supplementary file 3 — Supplementary Information3. [file 41598_2020_66926_MOESM3_ESM.tif]

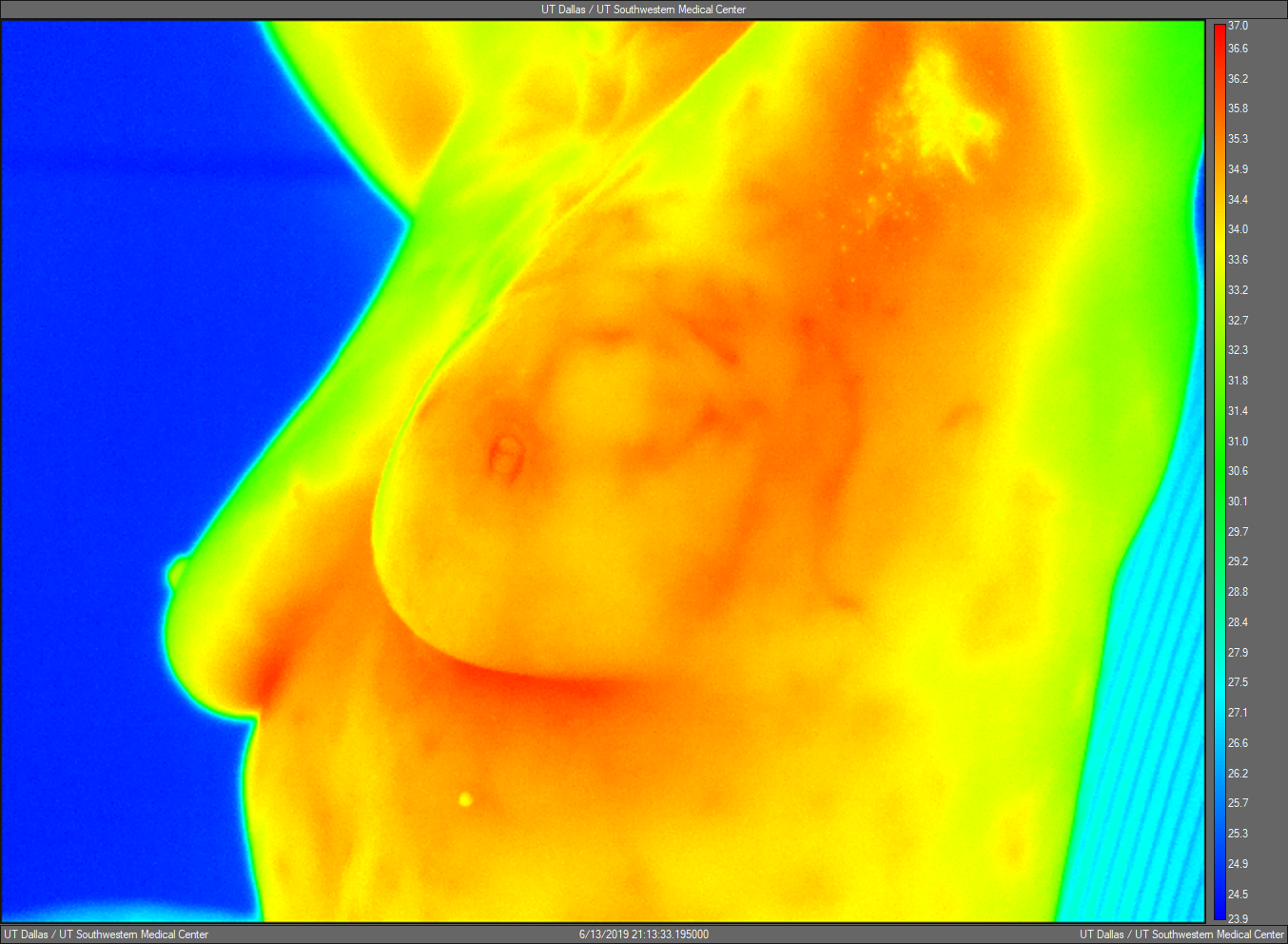

Supplement: Supplementary file 4 — Supplementary Information4. [file 41598_2020_66926_MOESM4_ESM.tif]

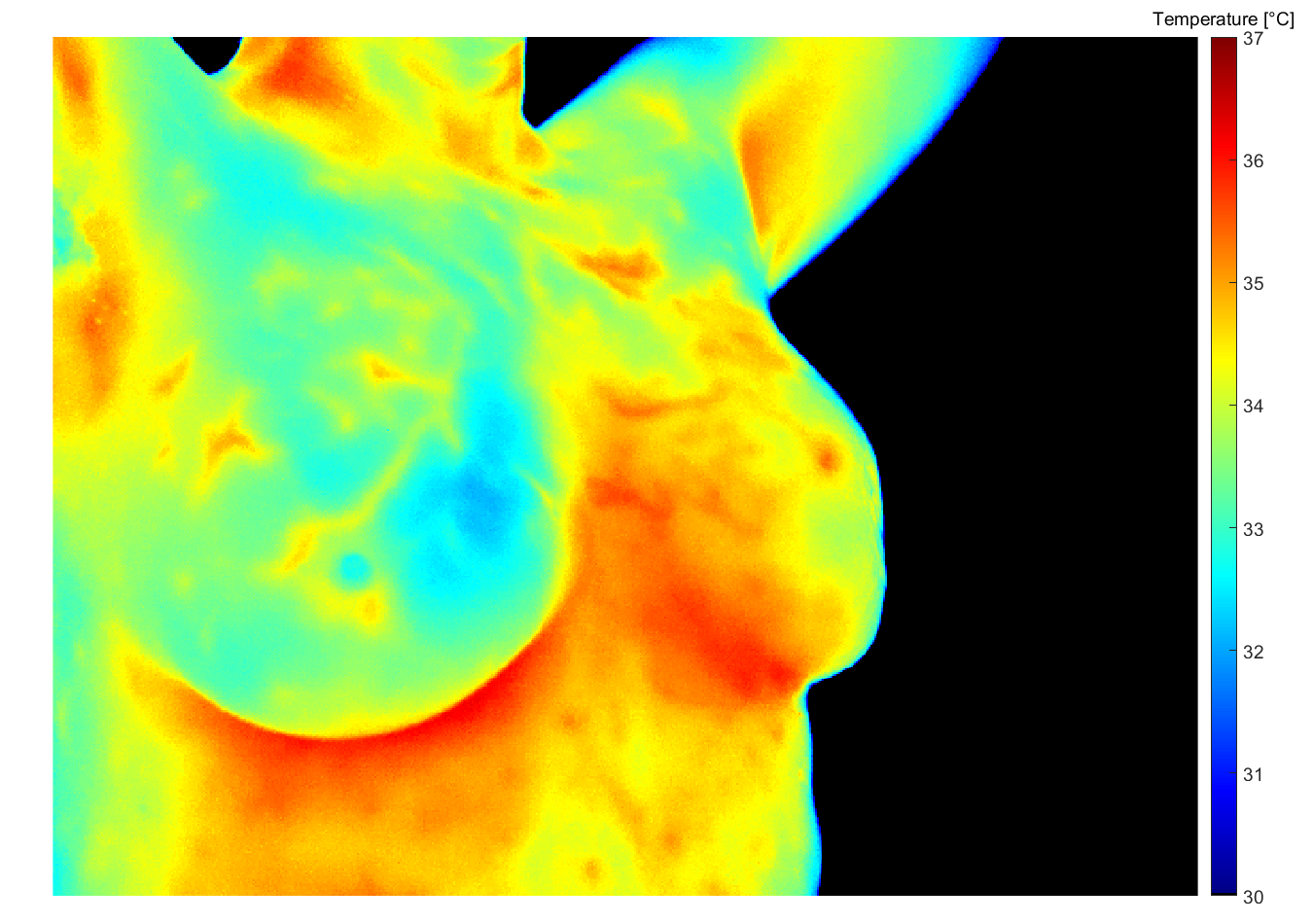

Supplement: Supplementary file 5 — Supplementary Information5. [file 41598_2020_66926_MOESM5_ESM.tif]

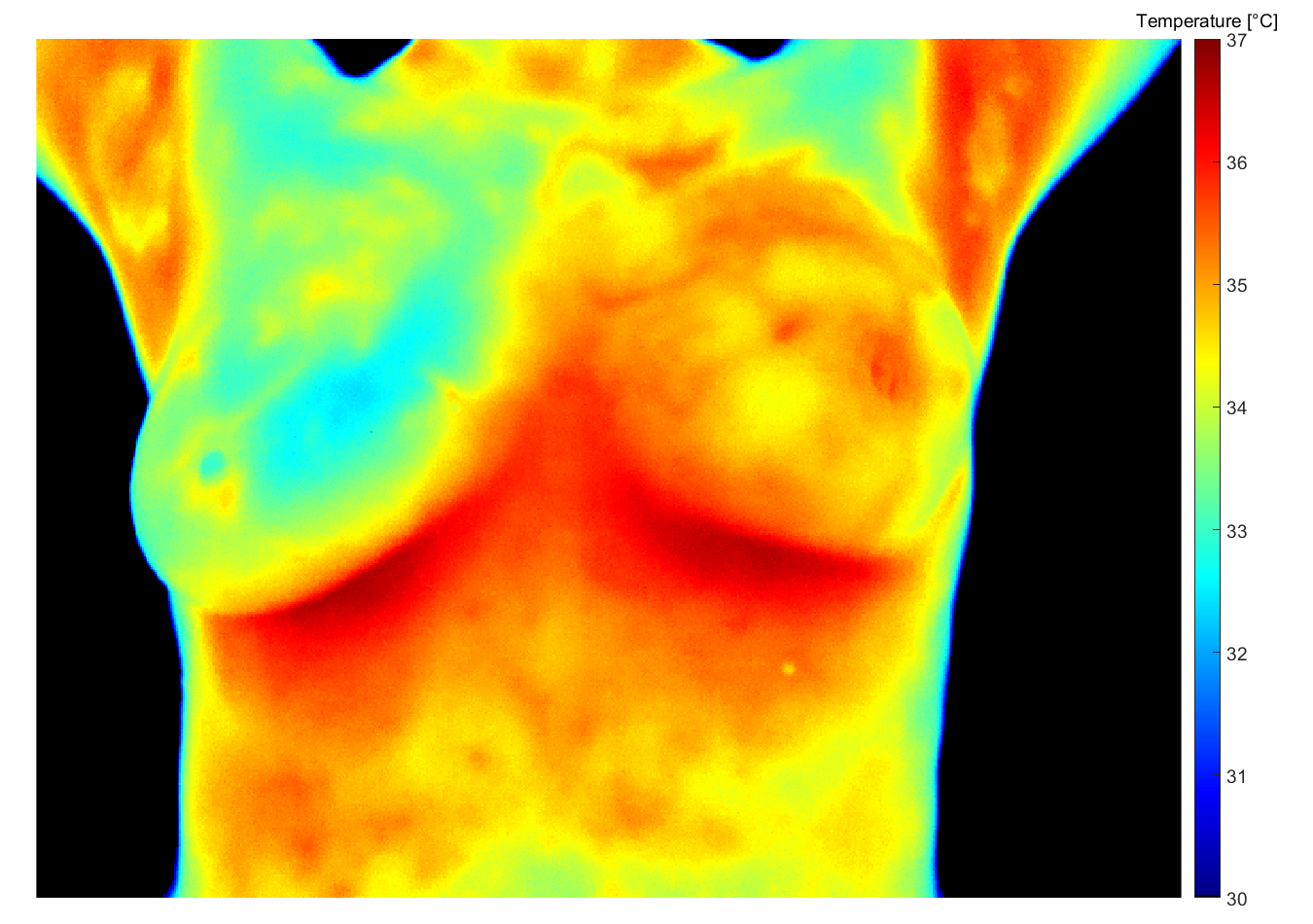

Supplement: Supplementary file 6 — Supplementary Information6. [file 41598_2020_66926_MOESM6_ESM.tif]

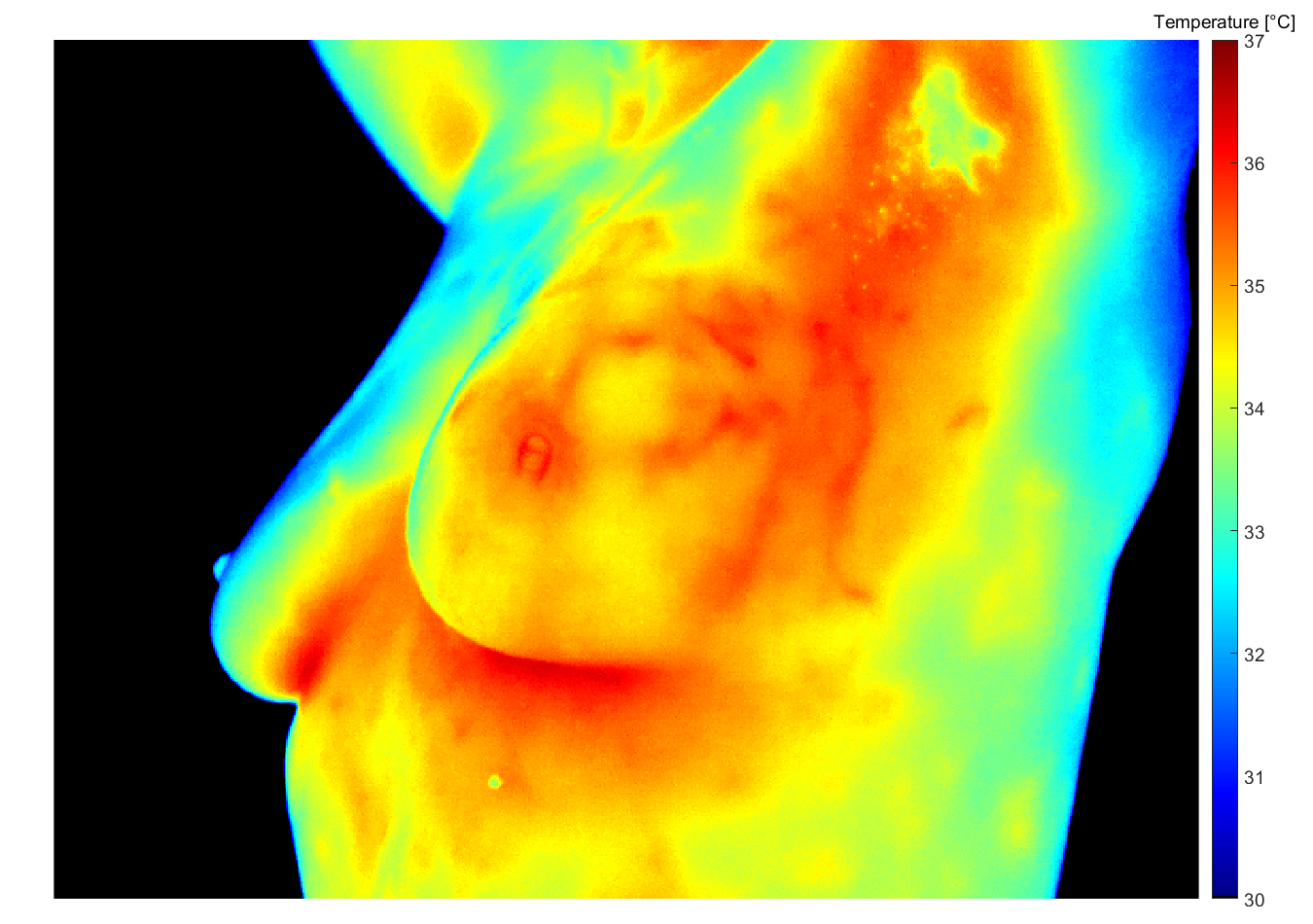

Supplement: Supplementary file 7 — Supplementary Information7. [file 41598_2020_66926_MOESM7_ESM.tif]
